# Skin cancer after heart transplantation: a systematic review^☆^

**DOI:** 10.1016/j.abd.2024.05.004

**Published:** 2024-11-16

**Authors:** Nathalia Hoffmann Guarda Aguzzoli, Ana Laura Bueno, Yağmur Halezeroğlu, Renan Rangel Bonamigo

**Affiliations:** a Dermatology Department, Universidade Federal de Ciências da Saúde de Porto Alegre, Porto Alegre, RS, Brazil; b Medical School Department, University of California, San Francisco, CA, United States; c Medical School Department, University of California Berkeley School of Public Health and University of California San Francisco School of Medicine, Berkely, CA, United States; d Dermatology Department, Universidade Federal do Rio Grande do Sul, Porto Alegre, RS, Brazil

**Keywords:** Heart transplantation, Immunosuppression therapy, Skin neoplasms

## Abstract

**Background:**

Cancer is an important cause of morbidity and mortality after solid organ transplants. Skin cancer is the most prevalent non-lymphoid malignancy occurring during heart transplantation follow-up. Due to the complexity of immunosuppressive therapy and the high prevalence and incidence of skin cancer in this population, dermatologists play an important role in the short and long-term follow-up of heart transplant recipients.

**Objectives:**

The goal of this study is to present data from a systematic literature review focusing on the occurrence of skin cancer in patients who have undergone heart transplantation.

**Methods:**

The authors conducted a systematic review of the literature in the EMBASE and PubMed databases from August to September 2021 to investigate the incidence of skin cancer in heart transplant patients. The authors selected retrospective and prospective cohort studies presenting data on the frequency of skin cancer in patients following heart transplantation. Exclusion criteria included articles that did not stratify the organ transplant type and studies that did not evaluate the frequency of skin cancer in the specific population.

**Results:**

Based on the search strategy, the authors found 2589 studies, out of which 37 were eligible for inclusion in this study. Provided data are from 20 different countries, over the period 1974 to 2015. Incidence of non-melanoma skin cancer (NMSC) ranges from 0.97% to 52.8%. The incidence of squamous cell carcinoma (SCC) ranges from 1.19% to 89% and the incidence of basal cell carcinoma (BCC) ranges from 2% to 63%. Malignant Melanoma (MM) incidence ranges from 0.94% to 4.6%

**Study limitations:**

The analysis involved an exclusive focus on heart transplant patients, and the statistical analysis of the sample may have been hampered. The significant heterogeneity among the studies emerged as a challenge during the analysis of the results. Furthermore, the study is limited by variations in follow-up periods among the included studies.

**Conclusion:**

Although gathering methodologically heterogeneous data, this systematic review was able to show the epidemiological importance of skin cancer in heart transplant patients. This study reinforces the important role dermatologists play in the short and long-term follow-up of heart transplant patients.

## Introduction

Malignancies are an important cause of morbidity and mortality after solid organ transplant.^1^ The correlation between immunosuppression and the development of malignancies is well documented.^2^, ^3^ Overall transplant recipients have an increased risk of developing a malignancy when compared with the general population, and heart transplants are described among those with the highest risk.^2^, ^3^ Malignancy incidences after heart transplantation are two to threefold higher compared to renal transplantation,^3^ and this fact can be attributed to the aggressive immunosuppression that these patients require.^3^, ^4^

Skin cancer is the most prevalent non-lymphoid malignancy occurring in heart transplantation follow-up.^5^ NMSC, such as Squamous Cell Carcinoma (SCC) and Basal Cell Carcinoma (BCC), are among the most prevalent.^2^, ^6^

The risk of development of SCC is known to be increased 10‒16 times in transplant patients compared to the general population.^6^ Studies estimate the incidence of SCC to be 65 to 250 times higher in Solid Organ Transplant Recipients (SOTRs) when compared with the general population.^7^ The incidence ratio of SCC to BCC in the general population is about 1:4. Conversely, among SOTRs, this ratio is 5:1, therefore SCC is more frequent than BCC in transplanted patients.^6^, ^8^

Melanoma is the most aggressive type of skin cancer and is also increased in SOTRs. The risk of developing melanoma after a solid organ transplant is two to eight-fold compared with the general population.^6^ Additionally, this population may have a worse melanoma prognosis.^9^

Despite the medication therapeutic arsenal, heart transplantation is a life-saving treatment in patients with advanced heart failure. It provides better survival rates than clinical therapy alone since advanced heart failure is a severe and life-threatening condition.^10^

The association of solid organ ttransplantsand skin cancer is well established, mainly in renal transplants.^6^ Currently, there are few studies focused specifically on patients with heart transplant and the development of skin tumors.

According to data from the Global Observatory on Donation and Transplantation (GODT), a total of 8408 heart transplantations were performed worldwide in 2021 across 54 countries. In the United States, based on information provided by the United Network for Organ Sharing (UNOS), there was a 4.3% increase in the percentage of heart transplantations between 2020 and 2021.^11^ This increase was maintained even during the COVID-19 pandemic.^11^

Considering the increased number of heart transplants performed in recent years, the improved long-term survival rates after this procedure, and the association of immunotherapy with malignancies, the authors believe that understanding thoroughly the epidemiology of skin cancer following heart transplantation is paramount to delivering adequate care for these patients and foster skin health equity.

The aim of this study is to present data from a systematic literature review on the presence of skin cancer among heart transplant patients.

## Materials and methods

### Search strategy

This study consisted of a systematic review of the literature on the frequency, prevalence, and incidence of skin cancer specifically among heart transplant patients.

The comprehensive search strategy was performed on PubMed on July 1^st^, 2021. Search in EMBASE and Scopus platforms was performed on August 27^th^, 2021. The authors used the following search strings to identify relevant papers: “*neoplasms*”, “*basal cell carcinoma*”, “*squamous cell*”, “*squamous cell carcinoma*”, “*nonmelanoma*”, “*melanoma*”, “*skin neoplasms*”, “*skin cancer*” and “*transplant recipient*”, “*organ transplantation*”, “*solid organ transplant*”, “*immunosuppression*”, “*immunocompromised host*” and “*heart*” or “*cardiac*”. The strategy also included searching for those terms in the bibliographic references of the selected articles.

### Selection of the studies

Selected articles were analyzed by two independent evaluators who critically reviewed the main characteristics of each study, such as population and sample size, casuistic of patients, country, dermatology evaluations and overall epidemiologic analysis. A third examiner evaluated and reviewed this work when disagreement was present.

Titles were read first, and then the titles and the abstracts. The articles that were within the scope of this research were read entirely. After reading the full text, the articles that met the inclusion criteria were selected. Studies that qualified for full-text revision were further analyzed by the following criteria: author, year of publication, study design, country, data/period evaluated, size of the study population, immunosuppressant medications, results, and other relevant information.

### Inclusion and exclusion criteria

The authors included original articles written in English concerning the prevalence and incidence of skin cancer in patients who received heart transplantation. The authors divided skin cancer into SCC, BCC and MM. In the selected articles, the frequency of skin cancer in the population studied had to be directly mentioned and easily calculated. The authors included retrospective and prospective cohort study designs in this analysis.

The exclusion criteria were review articles, clinical trial studies, articles with no individual evaluation per organ transplant type (evaluation only of the general cohorts), case reports, and studies that don't evaluate the frequency of skin cancer in the specific population. When institutions published duplicate cohort studies with accumulating numbers of patients, only the most complete reports were included.

### Quality assessment

The authors assessed the selected articles for methodological quality using the Newcastle Ottawa Scale (NOS) Quality Assessment Form for cohort studies. Studies with NOS scores 0–3, 4–6, and 7–9 were considered as low, moderate, and high quality, respectively.^12^

The authors add sun exposure estimate as a comparability variable criterion because it is an important risk factor to skin cancer development in any population. The authors considered the evaluation of the exposure to radiation UV in the questionnaire applied to the patients or the calculation of the time that this exposure represents.

The quality and relevance of the articles were investigated by two independent reviewers.

## Results

**Figure 1:**
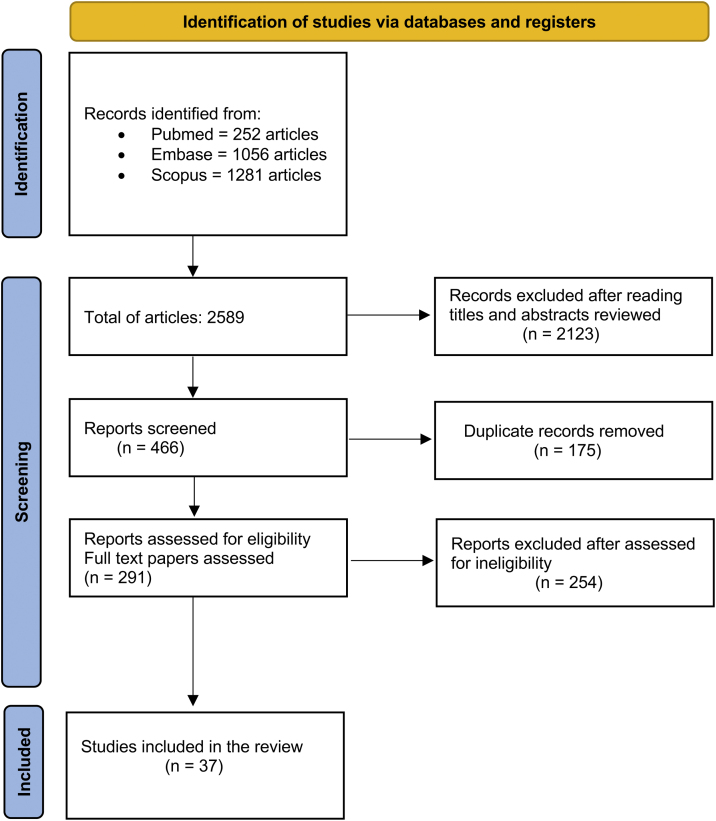
Search strategy.

A total of 2589 records were initially identified through the literature search. The authors performed the first screening by reading the titles. From the selected titles, the authors screened the abstracts, and 2123 articles were excluded in this process. The authors identified 175 duplicated articles. In the end, a total of 291 articles were read to the full extent and after screening for eligibility and inclusion criteria, 37 studies were included in this systematic review. Fig. 1 summarizes the search strategy and article selection. The selected articles and their clinical characteristics are summarized in Table 1.

**Table 1:** Summary of all the articles of the study.

First Author/ Year	Study Design	Country	Evaluated period	Sample Size (heart transplant) test	Immunosuppressant medications	Results: Skin Cancer in heart transplantation	Study design: prevalence or incidence and relevant other informations
Jensen et al. 1995^13^	Cohort	Norway	1983 to 1992 (9y)	140 (M: 113 F: 27) Mean Age^a^: 47.7 (2‒67)	CsA, AZA and prednisolone. No cytolytic induction therapy was used.	**Total of patients with Skin Cancer:** 18 (12.8%) **Total of skin lesions:** 27 lesions (0.19 lesions/patient) **SCC:** 10 (7.1%) **BCC:** 9 (6.4%) **MM:** 1 (0.7%) **Morbus Bowen:** 13 (9.3%) **Solar Keratosis (SK):** 18 **Keratoacantoma**: 8 (5.7%) None of the patients died of malignant skin tumors. No metastatic lesions were diagnosed.	Incidence
Espanã et al. 1995^14^	Cohort	Spain	1984 to 1993 (9y)	111 (M&F) Mean Age: 49.5 (2‒69)	1984 to 1986 and 1989 to 1993: CsA, 6-methylprednisolone and equine antithymocytic y-globulin. 1986 to 1989: CsA, AZA and corticosteroid. 1986 to 1989. In patients Cr >1.5, CsA was substituted for OKT3.	**Total of patients with Skin Cancer:** 14 (12.6%) **Total of skin lesions:** 26 (0.53 lesions/patient) **SCC:** 9-patients (64.2%) **BCC:** 8-patients (57.1%) **MM:** NA	Incidence SCC ratio was 1:1.5 for the first malignancy. BCC to SCC ratio was 1:1.3. Skin cancer appeared an average of 31.5-months after transplantation. BCC appeared at an average of 25.3-months, and the SCC appeared at 36-months. Most cancers appeared in the first 4-years after transplantation.
Sigfusson et al. 1996^15^	Cohort (Children)	United States	1975 to 1989 (14y)	68 (M&F) Mean Age: NA (<18y) Pediatric heart transplantation	1975 to 1980: AZA and corticosteroids 1980 to 1989: CsA, AZA and corticosteroids	**Total of patients with Skin Cancer:** 1 **Total of skin lesions:** 1 **SCC:** 1 **BCC: NA** **MM:** NA	Prevalence
Ong et al. 1999^16^	Cohort	Australia	1984 to 1998 (14y)	400 (M&F) Gender: NA Median age: 47.9 (6.6‒67)	AZA and CsA with or without prednisolone. Rejection episodes: IV pulsed methylprednisolone, OKT-3, antithymocyte globulin, total lymphoid irradiation, tacrolimus, MMF	**Total of patients with Skin Cancer:** 152 (38%) **Total of skin lesions:** 1436 **SCC:** 113 patients (28.2%) ‒ 849 lesions (2.1 lesions/patient) **BCC:** 92 patients (23%) ‒ 285 lesions (32.2%) **MM:** 7 patients (4.6%) ‒ 7 lesions **Actine Keratosis:** 19 patients ‒ 28 lesions **Bowen`s Disease:** 79‒263 lesions	Incidence Crude cumulative incidence rates of skin cancer: 1y: 8.5%; 3y: 21%; 5y: 31%; 10y: 43%. NSCC/BCC ratio of 3:1; a larger proportion of SCC in male than in female patients was found (3.2:1 vs. 1.3:1). Most lesions occurred on the head and neck. Metastases developed in 9 male patients with SCC and 4 with melanoma. Eleven deaths from skin cancer (6 from SCC, 4 from melanoma, 1 from Merkel cell carcinoma) accounted for 27% of deaths occurring after the fourth year after transplantation.
Fortina et al. 2000^17^	Cohort	Italy	Mean follow-up period: 4.7 years (1 month ‒ 12 years)	252 (M: 219 F:33) Mean age: 49 ± 14	CsA and AZA (double therapy, n = 67), or with CsA, AZA and oral prednisone (triple therapy, n = 185). Induction immunosuppression: CsA or of AZA administered 6 hours before operation, and a bolus of methylprednisolone during cardiopulmonary bypass. Rejection episodes: IV methylprednisolone, OKT3 or ATG.	**Total of patients with Skin Cancer:** 40 (15.8%) **Total of skin lesions:** 72 (1.8 lesions/patient) **SCC:** 17 patients (42%) ‒ 36 lesions. **BCC:** 20 patients (50%) ‒ 24 lesions **MM:** 1 patient (2.5%) ‒ 1 lesion **Bowen`s Disease:** 8 patients ‒ 9 lesions **Kaposi Sarcoma:** 1 **Merkeloma:** 1	Incidence SCC/BCC ratio in the transplant population was 1.17:1. A higher number of HT patients (40, 16%) developed at least One skin cancer compared to Kidney Transpant patients (16, 7%, p = 0.004). The mean interval between transplantation and detection of the first skin cancer was lower in HT (4.5 years) than in KT (6.7 years, p = 0.05).
Caforio et al. 2000^18^	Cohort	Italy	NA	300 (M:258 F:42) Mean age: 49 ± 15	Receiving standard double (CsA plus AZA) or triple (CsA plus AZA plus prednisone) therapy. HT recipients were treated with CsA and AZA (double therapy, n = 62) or with CsA, AZA and oral prednisone (triple therapy, n = 238). Postoperatively, the majority (83%) of patients received antilymphocyte globulin (ALG) or antithymocyte globulin (ATG), or both, for 3 to 5 days.	**Total of patients with Skin Cancer:** 48 (16%) **Total of skin lesions:** 104 (2.1 lesion/patient) **SCC:** 22 patients ‒ 53 lesions (45.8%) **BCC:** 24 patients (50%) ‒ 37 lesions **MM:** 2 patients (4.1%) ‒ 2 lesions **Bowen`s Disease:** 9 patients ‒ 9 lesions **Kaposi Sarcoma:** 1 patient ‒ 1 lesion **Merkeloma:** 1 patient ‒ 1 lesion	Incidence SCC/BCC ratio was 1.43:1. Large majority of lesions occurred on the head and neck (73, or 70%). Mean interval between HT and detection of the first skin cancer correlated with age at transplantation (p = 0.01). HT patients aged >50 years had an earlier onset of skin cancer lesions than those aged <50 (4 ± 3 vs. 6 ± 3 years, p < 0.001). 3 patients died of early metastasis of melanoma (n = 1), merkeloma (n = 1), and Kaposi’s sarcoma (n = 1).
Catena et al. 2001^19^	Cohort	Italy	Underwent transplantation until 1997	124 (M:124) Mean age: 55 (44 - 62)	All patients received triple immunosuppression, and all showed rejection episodes.	**Total of patients with Skin Cancer: NA** **Total of skin lesions:** NA **SCC: NA** **BCC: NA** **MM:** 2 (1.6%)	Incidence When cancer diagnosis was made, all patients had already metastatic disease. Marked disparity in the interval to De Novo Malignancy between KT and HT recipients (69 versus 24 months); this is probably related to the higher level of Immunosuppression therapy.
Caforio et al. 2001^20^	Cohort	Italy		304 (M: 261 F:43) Mean age: 49 ± 15	Standard double (CsA, AZA) or triple (CsA, AZA, prednisone therapy).	**Total of patients with Skin Cancer:** 57 (18.75%) **Total of skin lesions:** 104 lesions (1.82 lesions per patient) **SCC:** 26 patients (45.6%) **BCC:** 27 patients (47.3%) **MM: NA**	Incidence The SCC/BCC ratio was 1.43:1, and most lesions were on head and neck. HT > 49 years (p = .008; RR = 3.0), skin type II (p = .0001; RR = 3.5), solar keratosis (p = 0.0007, RR = 2.8), and sunlight exposure >30000 hours (p = 0.01; RR = 2.1) were risk factors for skin tumors of any type.
Fortina et al. 2004^21^	Cohort	Italy	At least 3 years of follow-up	230 (M: 198 F: 32) Mean age: 50.2 ± 11.0	CsA and AZA (double therapy; n = 37) or with CsA, AZA, and oral prednisone (triple therapy; n = 193). Oral prednisone was added to the double therapy regimen of CsA and AZA in cases of repeated or persistent rejection or of CsA nephrotoxic effects.	**Total of patients with Skin Cancer:** 48 patients (20.8%) **Total of skin lesions:** 120 lesions (just NMSC`s) **SCC:** 26 patients (54.1%) ‒ 83 lesions (3.19 lesions/patient) **BCC:** 13 patients (27%) ‒ 37 lesions (2.84 lesions/patient) **MM**: Not evaluated (NA)	Incidence **Cumulative incidence of NMSCs in HT recipients:** **SCC: 5y:** 6.5% (95% CI 3.2%‒9.7%); **10y:** 19.7% (95% CI 13.3%‒26.1%) **BCC: 5y:** 6% (95% CI, 2.9%‒9.1%); **10y:** 12% (7%‒17%) The cumulative risk of SCCs and BCCs increased steeply with increasing age at transplantation. The SCC/BCC ratio was 2.2:1. Most lesions occurred on the head and neck (n = 91; 69.5%). No metastatic or fatal tumors occurred. Median time between transplantation and appearance of the first NMSC was 5.2-years (3.5‒7.8 years, 25^th^‒75^th^ percentile).
Shiba et al. 2004^6^	Cohort	United States	1980 to 1991 (11y)	142 (M:109 F:33) Mean age: 51.4 ± 9.9	**Initial immunosuppressive regimens of 10-year survivors:** CsA/P/RATAG: 6 (4.2%); CsA/P: 23 (16.2%); CsA/AZA: 8 (5.6%); CsA/AZA/P/ATGAM: 12 (8.5%); CsA/AZA/P: 30 (21.1%); CsA/AZA/P/PKT3: 63 (44.4%); CsA was administered in all cases as part of the initial immunosuppressive regimen. **Maintenance regimens at 10 years included the following:** CsA AZA and prednisone: 70 (49.3%); CsA, AZA: 19 (13.4%) received; CsA, prednisone: 18 (12.7%); CsA, MMF and prednisone: 10 (7.0%); CsA and MMFl: 5 (3.5%); TCA and AZA: 2 (1.4%); TAC, MMF, and prednisone: 2 (1.4%); CsA only: 1 (0.7%); Unknown regimens: 14 (9.9%); Single drug regimen (only CsA): 2 (because of refractory malignancies.	**Total of patients with Skin cancer:** 34 (38%) **Total of skin lesions:** NA **SCC:** NA **BCC:** NA **MM**: 4 patients (2.8%) Fifty-four patients (38.0%) had skin cancers at 8.1 ± 3.7y (1.6–16.6 years) after HTx. Most lesions were recurrent squamous cell carcinoma or basal cell carcinoma	Incidence
El-hamamsy et al. 2005^22^	Cohort	Canada	1982 to 2002 (20y)	207 (M:172 F:35) Age: 46 ± 11	1988 and 2002: 154 patients received IV thymoglobuline. Oral prednisone, AZA or MMF, which has replaced AZA since 1997 were started after patient extubation. CsA was initiated on postoperative day 2 to day 5 after transplantation.	**Total of patients with Skin Cancer:** 84 patients (42%) **Total of skin lesions:** NA **Squamous Cell Carcinoma:** 1 patient (1.19%) **Basal Cell Carcinoma:** 16 patients (19.0%) **MM**: NA **Kaposi Sarcoma (KS):** 2% **Bowel:** 5%	Incidence Patients were divided into two groups: with a post-transplant neoplasm (n = 43) and cancer-free (n = 165). No differences between both groups regarding recipient age, gender, the underlying pathology, smoking history, and the number of treated rejection episodes in the first year. When examined separately, no significant association was found between the administration of any drug and the development of a neoplastic lesion.
Mello Júnior et al. 2006^23^	Cohort	Brazil	1986 to 2002 (16y)	106 (M:87 F:19) Mean age: 43.7 (12‒64)	All patients were submitted to CsA, AZA and a corticosteroid agent. Two patients also received orthoclone (OKT3) for treating rejection. The protocol of the immunosuppressive regimen consisted of CsA, AZA and corticosteroid.	**Total of patients with Skin Cancer:** 13 patients (12.26%) **Total of skin lesions:** NA **SCC:** 8 (34.80%) **BCC:** 5 (21.70%) **MM:** not avaliated (NA)	Incidence
Geusau et al. 2008^24^	Cohort	Austria	1984 to 2003 (19y)	322 (M:260 F:62) Median age: 54.02	Post-operatively: RATG, monoclonal murine antibodies muromonab OKT3 and monoclonal anti-IL-2 receptor antibodies. Maintenance therapy: triple drug regimen consisting of CsA or Tac; Aza or MMF, and prednisolone. From the beginning of 1998: all patients had been routinely switched from AZA to MMF.	**Total of patients with Skin Cancer:** 73 (23%) **Total of skin lesions:** 263 (3.6 lesions/patient) **SCC:** 27 patients (36.9%) ‒ 64 lesions (2.3 lesions/patient) **BCC:**46 patients (63%) ‒ 104 lesions (2.2 lesions/patient) **MM:** NA **Bowen`s Disease:** 25 patients (7.7%) ‒ 61 lesions (2.44 lesions/patient) **Bowen Carcinoma:** 16 patients (4.9%) ‒ 34 lesions (2.1 lesions/patient)	Incidence SCC (including BD and BowCa) to BCC ratio was 1.6:1. 33 patients had only a single skin tumor, 40 patients developed more than one tumor (28 had 2–5 skin malignancies, 12 more than 5). Cumulative incidence rates for NMSC: **5y:**7.3%; **10y:**26.9%; **15y:**56.5% Overall incidence rate of NMSC: 33.1 cases per 1000 post-transplant person- years. 32 of the patients (44%) with at least one tumor had AK (vs. 10% without tumor) (p < 0.0001). Average time from transplantation until diagnosis of the first NMSC: 79.56 months.
Brewer et al. 2009^25^	Cohort	United States	1988 to 2006 (18y)	All heart transplant recipients at Mayo Clinic from 1988 to 2006. (n = NA) 312 (M:227 F:85) had skin cancer. Mean age:47.4 ± 16.37	NA number of patients with the indivuadlly immunosuppressive therapy. Medications that were not significantly associated with the development of SCC included AZA, CsA, MMF, sirolimus, corticosteroids, and TAC. MMF was significantly associated with an increased risk of BCC (HR = 2.32; p = 0.005) Medications that were not significantly associated with the development of BCC included CsA, sirolimus, corticosteroids, and TAC. AZA was significantly associated with a decreased risk of BCC development (HR = 0.56; p < 0.05)	**Total of patients with Skin Cancer:** 312 **Total of skin lesions:** 1395 (9.6 lesions/patient) **SCC:** Patients NA ‒ 1236 lesions (89%) **BCC:** Patients NA ‒ 151 lesons (11%) **MM:** 5 lesions (<1%) **Angiosarcoma:** 1 patient **Atypical fibroxanthoma:** 1 patient **Pilomatrix carcinoma:** 1 patient	Incidence **Patients had an SCC:** **5y:**15.4% / **10y:**32.3% / **15y:**38.2% **Patients had an BCC:** **5y:**10.3% / **10y:**19.2% / **15y:**31.6% **Skin Cancer of any kind:** **5y:** 0.4% / **10y:** 7.5% / **15y:**46.4% **Cumulative incidence rates of a second SCC:** **1y:**44% / **3y:**67.4% / **5y:**75.9% **Cumulative incidence rates of having an SCC:** **1y:**36.7% / **3y:**54.7% / **5y:**65.9% **Cumulative incidence rates of a second BCC:** **1y:**32.1% / **3y:**48.6% / **5y:**51.4% **Cumulative incidence rates of a BCC developing:** **1y**: 16.3% / **3y**: 31.8% / **5y**: 45.7% **Cumulative Incidence of death:** **5y:** 18.4% (95% CI 13.6%‒23.0%); **10y:** 37.9% (95% CI 30.5%‒44.5%); **15y:** 63.5% (95% CI 51.5%‒72.5%); **18y:** 78.7% (95% CI 57.7%‒89.3%) Posttransplant SCC tumor burden of the 312 patients: 76 patients (24.4%) had at least 1 SCC, 24 patients (7.7%) had only 1 SCC, and 19 (6.1%) had 10 or more SCC. Evaluation of the BCC tumor burden of heart transplant recipients showed that 54 patients (17.3%) had at least 1 BCC, 23 patients (7.4%) had only 1 BCC, and 2 patients (0.6%) had 10 or more BCCs.
Chen et al. 2009^26^	Cohort	Taiwan	1987 to 2008 (21y)	66 (M&F)	All patients were treated with standard calcineurin inhibitor based triple immunosuppressive agent therapy (CsA or Tac; Aza or MMF, and prednisolone) After 1999, MMF gradually replaced AZA, and TAC replaced CsA as first-line immunosuppressive agents	**Total of patients with Skin Cancer:** 1 (1.5%) **Total of skin lesions:** 1 **SCC: 1** (1.5%) **BCC:** NA **MM:** NA	Incidence
Molina et al. 2010^27^	Cohort	Spain	1984 to 2003 (19)	3393 (M:2874 F:519) Mean age: 51.4 ± 11	**Patients receiving each kind of Immunosuppresor (percentages) by Period Post-HT (at any time):** CsA: 2863 (84.4%); AZA: 2334 (68.8%); Prednisone: 3365 (99.2%) TCA: 743 (21.9%); MMF: 1628 (48.0%); Sirolimus: 223 (6.6%); Everolimus: 27 (0.8%); OKT3: 1503 (44.3%); RATG: 580 (17.1%); Basiliximab: 227 (6.7%); Daclizumab:139 (4.1%)	**Total of patients with Skin Cancer:** 204 (6%) **Total of skin lesions:** 324 (1.58 lesion per patient) **SCC:** patients NA ‒ 169 lesions (52%) **BCC:** patients NA ‒ 104 lesions (32%) **MM:** patients NA ‒ 9 lesions (2.7%) **KS:** patients NA ‒ 4 lesions **Undifferentiated malignant tumors:** patients NA ‒ 23 lesions	Incidence RR of developing SCC in patients who were transplanted in a high sunshine zone (>2500 hours/year) was 8.7 (95% CI 4.3–17.8; p = .0001) in relation to patients who were transplanted in a low sunshine zone, and in BCC the RR was 3 (95% CI 1.7–5.4; p = 0.0001). AZA was associated with an increased SCC risk (RR = 1.8; 95% CI 1.2–2.7; p*=*0.003). Induction therapy was a risk factor for NMSC (RR = 2.1; 95% CI 1.6–2.7; p = 0.0001), SCC (RR = 2.3; 95% CI 1.6–3.4; p = 0.0001) and BCC (RR = 2.6; 95% CI 1.6–4.2; p = 0.0001), but only OKT3 was associated with both SCC and BCC.
Hsu et al. 2010 ^28^	Cohort	China	1987 to 2008 (21y)	291 (M:244 F:47) Mean age: 45.1 ± 16.1	All patients received triple drug immunosuppressive therapy. Since 1995, RTAG was used for induction therapy.	**Total of patients with Skin Cancer:** 3 (1%) **Total of skin lesions:** NA **SCC:** NA **BCC:** NA **MM:** patients NA ‒ 9 lesions (2.7%) No Kaposi’s sarcoma.	Incidence Incidence of skin cancers has slightly increased. It resulted probabily from a relative rarity of skin cancers in the Chinese population.
Doesch et al. 2010^29^	Cohort	Germany	1989 and 2005	211 (M:175 F:36) Mean age: 51.4 ± 10.5	All 211 transplant recipients received RATG intravenously. 1989‒2001: CsA combined with azathioprine 2001‒2003: CsA and MMF 2003 ‒ onward: mTOR inhibitors (everolimus/sirolimus)	**Total of patients with Skin Cancer:** 36 (17.1%) **SCC:** 9 patients (4.2%) - lesions NA **BCC:** 24 patients (11.3%) ‒ lesions NA **MM: NA** **Pre-cancerous lesions:** 3 patients (1.4%)	Incidence Mean interval from transplantation until initial diagnosis of a cutaneous malignancy was 7.6 ± 3.5 years.
Jensen et al. 2010^30^	Cohort	Denmark	1977 to 2006 (29y)	459 (M:368 F:91) Median age: 50 (2‒89)	High dose induction therapy up to one year after transplantation Maintenance therapy: AZA, CsA, prednisolone	**Total of patients with Skin Cancer:** 40 patients (8.7%) **Total of skin lesions:** NA **SCC:** 26 patients (65%) **BCC:** 14 patienst (35%) **MM:** NA	Incidence **Heart transplantation:** **BCC:** SIR of 5.6 (95% CI 3.1‒9.5); **SCC**: SIR of 113 (95% CI 74‒166) Highest risk of SCC among heart recipients, who are maintained on the highest dose regimen of immunosuppressive medication.
Hamour et al. 2011^10^	Cohort	England	1995 to 2007 (12y)	399 (M: 318 F:81) Mean age: 48 ± 12	All patients received CsA and corticosteroids with either MMF or AZA. Maintenance therapy: MMF and AZA Rejections: high-dose corticosteroids	**Total of patients with Skin Cancer:** NA **Total of skin lesions:** NA **SCC:** NA **BCC:** NA **MM:** NA	Incidence **Cumulative incidence of skin malignancy:** **1y=**2%**; 3y=**4%; **5y=**6%; **7y=**8%; **10y=**13%
Alam et al. 2011^31^	Cohort	United States	1993 to 2003 (10y)	6271 (M:4202 F:2069)	NA imunossupressive therapy protocol. Relative risk of skin cancer was 1.5 for higher dosages of CsA measured at the 1-year follow-up (6 vs. 2 mg/kg/d, p = 0.01), 1.4 for higher dosages of AZA (2.5 vs. 1 mg/kg/d, measured at 1-year follow-up, p = 0.007) and 1.4 for higher dosages of MMF (40 vs. 10 mg/kg/d, at one-year follow-up, p = 0.04).	**Total of patients with Skin Cancer:** 545 (8.6%) **Total of skin lesions:** NA **SCC:** 289 patients (53%) ‒ lesions NA **BCC:** 228 patients (41%) ‒ lesions NA **MM:** 22 patienst (4%) ‒ lesions NA **Other skin Cancer:** 6 patients	Incidence Lower latitude had a RR of skin cancer of 1.2 (p = 0.03). Pretransplantation history of skin cancer was associated with a 2.0 RR of skin cancer (p = 0.001). All-cause mortality after post-transplant skin cancer varied by type of skin cancer. For BCC, 5-year survival was 83% (95% CI 73–90%). For SCC, 5-year survival was 80% (95% CI 74–86%). For melanoma, 3-year survival was 50% (95% CI 27–73%). White patients were more susceptible to skin cancer than nonwhite patients (p < 0.0001), with 10-year freedom from skin cancer at 83% (95% CI 81–85%).
Chivukula et al. 2014^32^	Cohort	United States	2000 to 2011 (11y)	402 patients (M:310 F:92) Mean age: 54.0 ± 12.4	Subgroups: - 185 (46.0%) received alemtuzumab (M:140) - 56 (13.9%) thymoglobulin (M:46) - 161 (40.0%) no induction (M:124) 2000‒2006: calcineurin inhibitor and steroids typically 2006 ‒ onward: all patients received routine induction therapy with alemtuzumab and were subsequently treated with a CNI and a secondary agent with no postoperative steroid use.	**Alemtuzumab:** **Total of patients with Skin Cancer:** 15 (8.1%) ‒ lesion NA **SCC:** 10 patients (5.4%) ‒ lesion NA **BCC:** 4 patients (2.2%) ‒ lesion NA **MM:** 1 patient (0.5%) - lesion NA **Thymoglobulin:** **Total of patients with Skin Cancer:** 4 (71%) ‒ lesion NA **SCC:** 1 patient (1.8%) ‒ lesion NA **BCC:** 2 patients (3.6%) ‒ lesion NA **MM:** 1 (1.8%) ‒ lesion NA **No induction:** **Total of patients with Skin Cancer:** 11 (6.8%) ‒ lesion NA **SCC:** 5 patients (3.1%) ‒ lesion NA **BCC:** 5 patients (3.1%) ‒ lesion NA **MM:** 1 patient (0.6%) ‒ lesion NA	Incidence Skin cancers were the most common malignancies after cardiac transplantation: SCC followed by BCC. Neither alemtuzumab nor thymoglobulin was associated with enhanced rates of early skin malignancy. At 4 years after cardiac transplantation, induction with alemtuzumab showed similar rates of cancer-free survival, both overall and for nonskin cancers, compared with thymoglobulin and noninduced historical control subjects.
Fuchs et al. 2014^33^	Cohort	Germany	2003 to 2007 (4y)	145 (M&F) CSA group = mean age: 58.8 ± 11.4 TAC group ‒ mean age: 49.1 ± 13.0	Patients were divided into a CSA group (n = 25) and a TAC group (n = 120). Initial immunosuppressive therapy was performed in all patients with CSA, using AZA and methylprednisolone. Maintenance therapy: CSA or TAC or in combination with a DNA synthesis-inhibitor like AZA or MMF.	**Total of patients with Skin Cancer:** 7 (4.8%) **Total of skin lesions:** NA **SCC:** 2 patients (1.3%) ‒ lesions NA **BCC:** 3 patients (2%) ‒ lesions NA **MM:** 2 patients (1.3%) ‒ lesions NA	Incidence **Metastasis:** 1 malignant melanoma with metastases in lymph nodes. There was no significant difference between the 2 CNIs CSA and TAC regarding tumor incidence, overall survival, and important postoperative com- plications in HT patients.
Park et al. 2014^34^	Cohort	Korea	1990 to 2008 (18y)	207 (M&F)	NA imunossupressive therapy protocol.	**Total of patients with Skin Cancer:** 7 (3.3%) **Total of skin lesions:** NA **SCC:** NA **BCC:** NA **MM:** NA	Incidence Association between the indicated factors and the risk of skin cancer including carcinoma in situ in organ transplant recipients (heart only): Hazard ratio: 3.5 (1.3‒10.0) p = 0.01
Rivinius et al. 2015^3^	Cohort	Germany	1989 to 2014 (25y)	381 **(**M:300 F:81) Mean age: 51.2 ± 10.5	**Initial immunosuppressive:** CsA and AZA was replaced by CsA and MMF in 2001, and by TAC and MMF in 2006. mTOR inhibitors (everolimus/sirolimus) were used from 2003.	**Total of patients Skin Cancer:** 74 (19.4%) **SCC:** 28 patients (37,8%) - lesions NA **BCC:** 30 (40,6%) lesions NA **MM:** 2 (2.7%) ‒ lesions NA	Incidence
Secnikova et al. 2015^35^	Cohort	Czech Republic	1993 to 2010 (17y)	603 (M:493 F:110)	Standard initial regimen: steroids, calcineurine inhibitors (CsA or tacrolimus) and antimetabolites (MMF or AZA). 2004/2005 onward: mTOR inhibitors (sirolimus, everolimus)	**Total of patients with Skin Cancer:** 119 patients (19.7%) **Total of skin lesions:** NA **SCC:** 62 patients (52.1%) ‒ lesions NA **BCC:** 37 patients (31.1%) - lesions NA **MM:** 3 patients (2.5%) ‒ lesions NA **Actine Keratosis:** 11 patients (9.2%) ‒ lesions NA **Morbus Bowen:** 4 patients (3.3%) ‒ lesions NA **Merkell Cell Carcinoma:** 1 patient (0.84%) ‒ lesions NA	Incidence The median time to develop non-melanoma skin malignancy was 10 years.
Keer et al. 2016^4^	Cohort	Belgium	1987 to 2013 (26y)	541 (M:431 F:110) Mean age: 50 ± 14	Before 2000: CsA, AZA and methylprednisolone After 2000: MMF replaced AZA and AZA and TAC replaced CsA. All patients received induction therapy with polyclonal rabbit antithymocyte globulin. CsA: 272 (50%); TAC: 269 (50%); AZA: 237 (44%); MMF: 304 (56%)	**Total of patients with Skin Cancer:** 112 (20.7%) **Total of skin lesions:** 294 (2.6 lesions per patient) **SCC:** 58 patients (51.7%) ‒ lesions NA **BCC:** 51 patients (45.5%) ‒ lesions NA **MM:** 2 patients (1.7%) - lesions NA **KS:** 1 patient (0.89%) lesions NA	Incidence
Delgado et al. 2016^36^	Cohort	Spain	1984 to 2010	4561(M:3808 F:753) Mean Age: NA	The cohort was classified in PT ((PT/malignant non cardiac neoplasia): 77 (1.7%) and no previous tumor (NPT); 4484 (98.3%) with previous tumor. (No Previous Tumor group: 84% and Previous Tumor group: 62%)	**Total of patients with Skin Cancer:** 637 patients **Total of skin lesions:** NA **SCC:** 382 (PT = 8 NPT = 374) ‒ lesion NA **BCC:** 228 (PT = 2, NPT = 226) ‒ lesion NA **MM: 1**5 patients (PT = 2, NPT = 13) ‒ lesion NA **KS:** 12 (PT = 0 NPT = 12) ‒ lesion NP	Incidence This was the only study to report heart transplant recipients with previous neoplasia history
Mcpherson et al. 2017^37^	Cohort	Scotland	1992 to 2016 (24y)	363 (M:285; F:78)	Most common regime: CyA and MMF. 116 patients were given additional OKT3 induction between 1995 and 1998 and 72 patients were given r-ATG between 2010 and 2016 CyA + MMF: 216 patients; CyA + AZA: 111 patients	**Total of patients with Skin Cancer:** 60 (16.5%) **Total of skin lesions:** NA **SCC:** 26 (7.1%) ‒ lesion NA **BCC:** 34 (9.3%) ‒ lesion NA **MM:** NA	Incidence
Bhat et al. 2018^1^	Cohort	United States	1987 to 2015 (28y)	44.162 (M:33.767; F:10395) Mean age: 52.0 ± 12.0	91.4% of HT recipients were using 3 or more immunosuppressant medications.	**Total of patients Skin Cancer:** 5060 (11.4%) **Total of skin lesions:** NA **SCC:** 3218 patients (63.5%) ‒ lesion NA **BCC:** 1175 patients (23.2%) ‒ lesion NA **MM:** 48 (0.94%) ‒ lesion NA **KS:** 19 (0.37%) ‒ lesion NA	Incidence **Cumulative Incidence of Cancer at Various Time Points: Heart (% [95% CI])** 1y = 1.4 (1.3‒1.5); 5y = 10 (9.6‒10.3); 10y = 21 (20.5‒21.4); 15y = 28.2 (27.5‒28.7); 20y = 32 (31.3‒32.6) Duration of follow up (y), mean ± SD (range): 5.8 (0‒24.3)
Jäämaa-Holmberg et al. 2019^38^	Cohort	Finland	1985 to 2014 (29y)	479 (M:381; F:98) Median age: 52	All patients: received immunosuppression based on calcineurin inhibitors (CsA or TAC) combined with either AZA or MMF Until the end of 2010: polyclonal anti-thymocyte antibodies were administered during the first three days for all patients.	**Total of patients Skin Cancer:** 145 (30.2%) **Total of skin lesions:** NA **SCC:** 56 patients (38.6%) ‒ lesion NA **BCC:** 83 patients (57.2%) ‒ lesion NA **MM:** 5 patients (3.4%) ‒ lesion NA **KS:** 1 patient (0.68%) ‒ lesion NA	Incidence **Expected / Standardized Incidence ratio / 95%CI / p-value** **Squamous Cell Carcinoma:** 1.1 / 51.9 / 39.2‒67.4 / p ≤ 0.001 **Basal Cell Carcinoma:** 7.9/10.5 / 8.4‒13.0 / p ≤ 0.001 **Melanoma:** 1.3 / 3.8 / 1.2‒8.8 / p ≤ 0.01 **Kaposi's Sarcoma:** 0 / 365.0 / 9.2‒2033 / p ≤ 0.01
Kimura et al. 2019^39^	Cohort	Japan	1999 to 2017 (18y)	103 (M:79; F:24) Malignancy group (n = 7) and a no-malignancy group (n = 96). Mean age: 39.6 ± 12.6	**Initial maintenance therapy** **Prednisolone:** 103 (100%) **CsA:** 20 (19%) / **TAC:**83 (81%) / **MMF:** 100 (97%) / **AZA:** 2 (1.9%) / **Sirolimus:** 1 (1%) **Maintenance therapy at the end point:** **Prednisolone:** 27 (26%) / **CsA:** 8 (7.8%) / **TAC:** 88 (85%) / **MMF:** 38 (37%) / **Everolimus:** 67 (65%) **Induction therapy:** 41 (40%) **Basiliximab:** 33 (32%) / **OKT3:** 7 (6.8%) / **Daclizumab:** 1 (1%)	**Total of patients with Skin Cancer:** 1 patient (0.97%) **Bowen's disease:** 1 patient **SCC:** NA **BCC:** NA **MM:** NA	Incidence PTLD and colon cancer were more common than skin cancer in Japanese recipients. These differences might be caused by differences in ethnicity, diet, environment, and viral status. For example, skin type II and sunlight exposure >30,000 h, are not common in Japan and may explain the low prevalence of skin cancer.
Park et al. 2019^40^	Cohort	Canadá	1994 to 2013 (19y)	684 (M:530; F:144) Median age: 53 (44‒59)	NA	**Total of patients with Skin Cancer:** NA **Bowen's disease:** NA **SCC:** NA **BCC:** NA **MM:** NA	Incidence **Cumulative incidence of Keratinocyte Carcinoma (95% CI) ‒ heart transplantation** **2y:** 5.68 (4.10‒7.61) **5y:** 15.04 (12.32‒18.02) **10y:** 26.67 (22.73‒30.76) **15y:** 34.27 (29.37‒39.23) **19y:** 37.18 (31.48‒42.87)
Asleh et al. 2019^41^	Cohort	United States	1994 to 2016 (22y)	523 (M:354; F:169) Mean age: 50.0 ± 13.6	All patients received induction therapy with RATG, and a minority of patients received muromonab-CD3 (OKT3) Maintenance: CNI (tacrolimus or cyclosporine), an antimetabolite (MMF or AZA), and tapering doses of prednisone.	**Distribution of Cancer Events While on CNI or SRL Therapy** **SCC:** **Overall:** 123 (23.5%) / **While on CNI:** 70 (13.3%) / **While on SRL:** 53 (10.1%) **BCC:** **Overall:** 46 (8.7%) / **While on CNI:** 22 (4.2%) / **While on SRL:** 24 (4.5%) **Total number of subsequent primary occurrences of NMSC** **SCC:** **Overall:** 276 (52.7%)/ **While on CNI:** 179 (34.2%) **/ While on SRL:** 97 (18.5%) **BCC:** **Overall:** 41 (7.8%) / **While on CNI:** 19 **(3.6%) / While on SRL:** 2 (0.3%) **MM:** NA	Incidence Most malignancies were NMSCs (n = 169; 92 in the CNI and 77 in the SRL groups). SRL conversion was associated with a significantly decreased risk of subsequent primary occurrences of NMSC compared with CNI therapy (unadjusted HR = 0.44; 95% CI: 0.27 to 0.71; p < 0.001; adjusted HR = 0.44; 95% CI 0.28 to 0.69; p < 0.001)
O'neill et al. 2019^42^	Cohort	Ireland	1994 to 2014 (20y)	214 (M:166; F:48) Median age: 47.1	‒	**Total of patients with Skin Cancer:** 59 patients (27%) **Total of skin lesions:** NA **SCC:** 36 patients (16.8%) **BCC:** 40 patients (18.6%) **MM:** 0	Incidence Standardized incidence ratios (**SIR**) for All Skin in Heart transplant: 9.26 (7.05‒11.94) Total Incident cases: 59 **SIR** for NMSC (keratinocyte carcinoma) in Heart transplant: 9.87 (7.51‒12.73) Total Incident cases: 59 **SRI** for NMSC BCC in Heart transplant: 8.7 (6.22‒11.85) Total Incident cases: 40 **SRI** for NMSC SCC in Heart transplant:19.05 (13.34‒26.37) Total Incident cases: 36
Infusino et al. 2020^43^	Cohort	Italy	1974 to 2014 (40y)	133 (M&F)	Patients were treated with immunosuppressant drugs in accordance with the specific guidelines Alone or in association, cyclosporine, tacrolimus or sirolimus, while sodium mycophenolate, mycophenolate mofetil and everolimus were always administered in association with other drugs.	**Total of patients with Skin Cancer:** 70 (52.6%) **Total of skin lesions:** NA **SCC + Keratosis Actinic:** 91 (68.4%) **BCC:** 21 (15.7%) **MM:** 0	Incidence The heart transplant patients showed statistically significant higher rates of nmSC and aK compared with other organ transplants (52.6%, p = 0.0352).
Yeh et al. 2020^44^	Cohort	Taiwan	1997 to 2011 (14y)	687 (M:548; F:139)	**TCA:** 188 (27.4%); **CSA:** 445 (64.3%); **MMF:** 433 (63.0%); **AZA:** 111 (16.2) **Everolimus:** 47 (6.8%) **Sirolimus:** 0 (0%) **Steroid: 638** (92.9%) Approximately 79% of heart transplant recipients used thymoglobulin	**Total of patients with Skin Cancer:** 2 (0.29%) **Total of skin lesions:** NA **SCC:** NA **BCC:** NA **MM:** 0	Incidence Significantly higher incidences of nonmelanoma skin cancer SIR (95% CI): 5.8 (1.5‒23.3) **Standardized incidence ratio** Significantly higher incidences of nonmelanoma skin cancer (SIR = 5.8; 95% CI, 1.5‒23.3; p < 0.05)

AK, Actinic Keratosis; AZA, Azathioprine; BCC, Basal Cell Carcinoma; CNI, Calcinerin Inhibitor; CsA, Cyclosporine; HT/HTx, Heart Transplant; KT, Kidney Transplant; MM, Malignant Melanoma; MMF, Mycophenolate Mofetil; mTOR, Mammalian Target of rapamycin; NMSCR, Non-Melanoma Skin Cancer; NA, Not Available; NPT, No Previous Tumor; RATG, Rabbit Antithymocyte immunoglobulin; SCCI, Squamous Cell Carcinoma; SRL, Sirolimus; TAC, Tacrolimus; M&F, Male and Female.

a Age unit in years; CI, Confidence Interval; RR, Relative Risk.

The 37 studies included in this review, they were conducted in 19 different countries: United States 7 studies, Italy 6 studies, Germany 3 studies, Spain 3 studies, Taiwan 2 studies, Australia 2 studies, Canada 2 studies, Belgium 1 study, Japan 1 study, Finland 1 study, Brazil 1 study, England 1 study, Norway 1 study, Korea 1 study, China 1 study, Ireland 1 study, Czech Republic 1 study, Denmark 1 study, Scotland 1 study. Provided data are from 19 different countries, over the period of 1974 to 2015.

The methodological quality of the studies varied with a range of scores between 1‒9, using the tool Newcastle Ottawa Scale (NOS) Quality Assessment Form for cohort studies. Table 2 shows the quality control for each of the selected articles. In the evaluation, 23 (62%) studies were classified as good quality, 2 (5,4%) studies were classified as fair quality and 12 (32,4%) studies were classified as poor quality.

**Table 2:** Summary of the quality analysis using the Newcastle-Ottawa Quality Assessment Form for Cohort Studies modified.

Study	Selection				Comparability		Outcome			Quality
	Representativeness of cohort	Selection of the non-exposed cohort	Ascertainment of exposure	Demonstration that outcome of interest was not present at start of study	Comparability of cohorts on the basis of the design or analysis		Assessment of outcome	Was follow-up long enough for outcomes to occur	Adequacy of follow up of cohorts	
					Age and Gender	Sun exposure estimate				
Jensen 1995	⋆	‒	⋆	⋆	‒	‒	⋆	⋆	⋆	
España 1995	⋆	‒	⋆	⋆	‒	‒	⋆	⋆	⋆	
Sigfusson 1996	⋆	‒	⋆	⋆	⋆	‒	⋆	⋆	⋆	
Ong 1999	⋆	‒	⋆	⋆	⋆	‒	⋆	⋆	⋆	
Fortina 2000	⋆	⋆	⋆	⋆	⋆	⋆	⋆	⋆	⋆	
Caforio 2000	⋆	‒	‒	⋆	⋆	⋆	⋆	⋆	⋆	
Catena 2001	⋆	‒	⋆	⋆	‒	‒	⋆	⋆	⋆	
Caforio 2001	⋆	‒	‒	⋆	‒	‒	‒	⋆	⋆	
Fortina 2004	⋆	‒	‒	⋆	⋆	⋆	⋆	⋆	⋆	
Shiba 2004	⋆	‒	⋆	⋆	‒	‒	⋆	⋆	⋆	
El-hamamsy 2005	⋆	⋆	⋆	⋆	⋆	‒	⋆	⋆	⋆	
Mello Júnior et al. 2006	⋆	‒	⋆	⋆	‒	‒	⋆	⋆	⋆	
Geusau 2008	⋆	‒	⋆	⋆	⋆	⋆	⋆	⋆	⋆	
Brewer 2009	⋆	‒	⋆	⋆	⋆	‒	⋆	⋆	⋆	
Chen 2009	⋆	‒	⋆	⋆	‒	‒	⋆	⋆	⋆	
Molina 2010	⋆	‒	⋆	⋆	⋆	⋆	⋆	⋆	⋆	
Hsu 2010	⋆	‒	⋆	⋆	⋆	‒	⋆	⋆	⋆	
Doesch 2010	⋆	‒	⋆	⋆	⋆	‒	⋆	⋆	⋆	
Jensen 2010	⋆	‒	⋆	⋆	⋆	‒	⋆	⋆	⋆	
Hamour 2011	⋆	‒	⋆	⋆	-	‒	⋆	⋆	⋆	
Alam 2011	⋆	‒	⋆	⋆	-	‒	⋆	⋆	⋆	
Chivukula 2014	⋆	⋆	⋆	⋆	⋆	‒	⋆	⋆	⋆	
Fuchs 2014	⋆	‒	⋆	⋆	⋆	‒	⋆	⋆	⋆	
Park 2014	⋆	‒	⋆	⋆	⋆	‒	⋆	⋆	⋆	
Rivinius 2015	⋆	‒	⋆	⋆	⋆	‒	⋆	⋆	⋆	
Secnikova 2015	⋆	‒	⋆	⋆	⋆	‒	⋆	⋆	⋆	
Keer 2016	⋆	‒	⋆	⋆		‒	⋆	⋆	⋆	
Delgado 2016	⋆	⋆	⋆	⋆	⋆	‒	⋆	⋆	⋆	
Mcpherson 2017	⋆	‒	⋆	⋆	‒	‒	⋆	⋆	⋆	
Bhad 2018	⋆	-	⋆	⋆	⋆	‒	⋆	⋆	⋆	
Jäämaa-Holmberg 2019	⋆	-	⋆	⋆		‒	⋆	⋆	⋆	
Kimura 2019	⋆	‒	⋆	⋆	⋆	‒	⋆	⋆	⋆	
Park 2019	⋆	‒	⋆	⋆	⋆	‒	⋆	⋆	⋆	
Asleh 2019	⋆	‒	⋆	⋆	⋆	‒	⋆	⋆	⋆	
O'neill et al. 2019	⋆	‒	⋆	⋆	⋆	‒	⋆	⋆	⋆	
Infusino 2020	⋆	‒	⋆	⋆	‒	‒	⋆	⋆	⋆	
Yeh 2020	⋆	‒	⋆	⋆	⋆	‒	⋆	⋆	⋆	

Legend: Quality: Good Quality (); Fair Quality (); Poor Quality ().

Good quality, 3 or 4 stars in selection domain and 1 or 2 stars in comparability domain and 2 or 3 stars in outcome/exposure domain. Fair quality, 2 stars in selection domain and 1 or 2 stars in comparability domain and 2 or 3 stars in outcome/exposure domain. Poor quality, 0 or 1 star in selection domain or 0 stars in comparability domain or 0 or 1 stars in outcome/exposure domain.

Herein the authors provide a comprehensive overview of the frequency of skin cancer among patients following heart transplantation.

## Discussion

The authors conducted a comprehensive systematic review of skin cancer among patients who underwent heart transplantation, summarizing data from 37 studies. To the author’s knowledge, this is the first systematic review focusing specifically on the frequency of skin cancer in this specific population of patients.

The incidence of NMSC in this systematic review ranges from 0.97% in a cohort from Taiwan to 52.8% in an Italian cohort.

The highest incidence of NMSC in the Italy study (Infusino et al. 2020) could possibly be attributed to the good size of the sample, long-term follow-up, analysis performed by type of organ transplant, predisposition of white skin in Italian patients, UV light exposure, and high immunosuppression prescription among heart transplant patients.^43^

The lowest incidence of NMSC in Taiwan (Yeh et al.) could be influenced by genetic factors that lower the prevalence of such cancer in the Asian population.^44^

The incidence of SCC ranges from 1.19% in a study conducted in Canada to 89% in a study conducted in the United States.^22^, ^25^ The incidence of BCC varies between 2% to 63%, in Germany and Austria, respectively.^24^, ^33^ It is in accordance with the existing literature, it is observed that SCC occurs more frequently than BCC in organ solid recipients.

Considering MM, the highest incidence was 4.6% in a study conducted in Australia (Ong et al.).^16^ This study confirms significant associations between skin cancer and HLA antigens among heart transplant patients. It also reinforces the importance of regular skin examinations in the follow-up.

The lowest incidence of MM was 0.94% in a cohort from the United States (Bhat et al.).^1^ Limitations of this study include the underreporting of skin cancers in the registry and individual risk factors for skin cancer.

All articles that provide the gender on the demographic characteristics of the sample had a predominance of males in the population in the analysis.

Skin cancer is the most common malignancy among solid organ transplant recipients. Among the skin cancer subtypes, non-melanoma is the most prevalent in this population. It is also well established that heart transplant patients require higher doses of immunosuppressive therapy before and after the surgical procedure in comparison to other solid organ transplantation.

Many studies from the literature have addressed the prevalence of skin cancer in kidney transplantation follow-up. Although it is a more common procedure than a heart transplant, patients generally need lower doses of immunosuppressive therapy following the procedure. Therefore, it is paramount to understand the effect of immunosuppressive therapy in different solid organ transplants on the outcome of skin cancer.

Several factors contribute to the development of malignancies after transplantation, including older age at transplantation, retransplantation, type and degree of immunosuppression therapy, sunlight exposure, skin type, and male sex.^1^, ^17^, ^25^, ^27^^,^^45^

Accordingly, the authors highlight that heart transplant patients are particularly susceptible to developing skin cancer because two of main factors: the intense immunosuppression required, as well as older age at the time of the transplant.^1^, ^25^

Furthermore, the authors emphasize that beyond its high prevalence, SCC appears to be more aggressive in Solid Organ Recipient Transplants (SORTs), with a higher risk of metastasis. These patients are on average 10 times at a greater risk of metastasis when compared to the general population, which is associated with increased morbidity and mortality of these patients.^5^, ^18^, ^46^ To explore a comprehensive understanding of the behavior of SCC in patients with heightened immunosuppression, such as heart transplant patients, is a crucial point.

Solid organ transplant recipients have an increased risk of developing malignant melanoma.^6^, ^46^ It is estimated that they have up to 2‒8-fold increased risk compared to the general population, with an average time to development of 5-years post-transplant.^6^, ^46^ Studies show that the likelihood of developing melanoma after heart transplantation is higher when compared with other organ transplant recipient subgroups, which can be attributed to more intensive immunosuppressive therapy in those patients.^47^

Although, these data are controversial in the literature since some studies don’t show a substantial risk of melanoma among these patients.^48^ As there are many articles showing an increased risk of melanoma in SORTs, and considering that melanoma is a potentially fatal malignancy, the authors recommended that healthcare providers need to proceed with caution considering MM between vulnerable immunosuppressive populations.^6^, ^9^, ^46^, ^47^^,^^49^ Another important fact to highlight in this context is that the incidence of MM has been increasing in the general population, which should be an alarm to susceptible groups, such as the population in this study.^9^, ^47^, ^50^

In this systematic review, just one study analyzed the pediatric heart transplant population. Sigfússon et al evaluated all the heart transplants in a small center in patients under the age of 18, in a total of 68 patients that survive 5-years after the procedure, only 1 SCC was registered.^15^

Most of the articles provide the mean age at the time of the transplantation and the standard deviation. Many articles do not provide the range of age, so the authors cannot determine which age is the youngest in the cohort.

The present review is in accordance with the literature regarding skin cancer as the most common malignancy between solid organ transplants. Associated with this fact, among solid organ transplants, patients with heart transplants are considered a vulnerable population because they need intense and strong immunosuppressive therapy. Since the risk of rejection of the transplanted organ can be fatal, these patients usually receive 3 different types of immunosuppressive drugs. This fact can be one of the main reasons why these patients have even higher rates of skin cancer, as this study suggests. This review shows that most of the studies found an increased frequency of skin cancer in this population and shows the importance of target studies focused on this specific population.

Interestingly, the present analysis confirms that the incidence of skin cancer is reported to be lower in Asian countries, including studies in Taiwan, Japan, Korea, and China.^26^, ^28^, ^34^, ^39^^,^^44^ These authors suggest that genetic influence, ethnicity, diet, environment, viral status, phototype, and sunlight exposure could justify this observed phenomenon.^26^, ^28^, ^39^, ^40^^,^^44^

Generally, it was difficult to determine if the skin neoplasms existed before the transplant or if these lesions developed after the immunosuppression therapy, because this information is not provided by the papers. The patients were not examined by a dermatologist before the heart transplantation, so there is no data to support that pre-existing lesions were absent and that all the skin cancer was a consequence of the immunosuppressive therapy, although the authors presume it is probably true for most of the cases. A thorough dermatologic evaluation prior to the solid organ transplant is also important because one of the difficulties was to establish the correct classification of terms: incidence, prevalence, and frequency rates.

### Immunosuppression therapy

Focusing on the immunosuppressive medications in heart transplant and skin cancer, Bhat et al. 2018 show that a lower number of immunosuppression medications is associated with a decreased incidence of skin cancer, and no significant changes were found for Kaposi's Sarcoma.^1^

Fortina et al. in 2000 et al. found that the type of immunosuppressive regimen did not affect the risk of skin cancer in heart transplant patients.^17^ In another study in 2004, they found no association between cumulative doses of each single immunosuppressive drug used in the maintenance or acute rejection immunosuppression, and the risk of development of BCC`s and SCC`s, in heart transplant patients.^21^ They also suggest that SCC`s, but not BCC`s, are related to global immunosuppression levels after 3 years rather than to specific immunosuppressive drugs. Another point in this study is that SCC was increased substantially by high occupational sunlight exposure, but not BCC.^21^

The results of Molina et al. 2010, suggest that SCC in heart transplants depends on the type of immunosuppressive therapy that is prescribed, and not just the duration and the dose of the immunosuppression.^27^ They found that Mycophenolate mofetil; was a protective factor against SCC, azathioprine was a risk factor, and tacrolimus and cyclosporine had no effect.^27^ In this study, no immunosuppressive drugs were associated with BCC and high sunlight exposure was a risk factor for both tumors, BCC and SCC.^27^

Brewer et al. 2009 show an increased risk of BCC in patients using MMF compared to those using Azathioprine, while tacrolimus and sirolimus had no significant effects to decrease the risk of BCC and SCC, respectively.^25^ Asleh et al. 2019 show that the substitution of calcineurin inhibitor by sirolimus had a positive impact on susceptible patients with non-melanoma skin cancer history in the post-heart transplant period.^41^ Fuchs et al. 2014, in a comparison study between cyclosporine and tacrolimus after a heart transplant, did not see significant differences between the groups, including skin cancer analysis.^33^

Geusau et al. 2008 found that an increased number of NMSC correlates with the duration of the immunosuppressive therapy, and regimens containing Azathioprine seemed influential on the development of NMSC, however, this effect was not statistically significant.^24^ This group concluded that the induction therapy does not appear to be associated with higher incidences of NMSC.^24^

Many studies suggest that mTOR may have a slightly protective effect in preventing skin cancer in solid organ transplants.^1^, ^3^, ^51^

During this review, drawing conclusions about the association between immunosuppressive therapy and skin cancer was a challenge, because most of the studies included patients with solid organ transplant in general, and they didn't evaluate organs, immunosuppression therapy and malignancies separately.

Clearly, the authors see the importance and the necessity of an evaluation of the heart transplant patients, focusing only on the skin cancer outcome and the association of this outcome with the induction and maintenance immunosuppression therapy.

Furthermore, the authors need to better characterize the correlation between immunosuppressive therapy and the other risk factors for skin cancer, which is complex but necessary to evaluate individually in each patient. The authors believe this gap in knowledge needs to be addressed to advance precision medicine in this specific population. Although skin cancer is the most common malignancy in transplant patients, and it influences directly mortality and morbidity rates, the authors emphasize the necessity of an accurate evaluation of this outcome.

### Limitations of the study

The present study has some limitations. First, most of the studies are cohorts of patients who underwent a solid organ transplant. They analyzed all the cohorts, and they didn't focus specifically on heart transplantation and its peculiarities. This can be a risk of bias because the heart transplant population has unique features. The authors selected the analysis that involved only heart transplant patients. In this way, statistical analysis of the sample may have been hampered.

Difficulties in the analysis occurred, mainly due to the heterogeneity of the studies; the authors reinforce this point because it was the most notable challenge during the analysis of the result. The studies are heterogeneous in organizing the statistical analysis. Moreover, immunosuppression protocols vary according to the different centers and protocols.

The authors also highlight that the study design of an authentic cohort study is compromised when there is not a comparison cohort, which derives from the same center, and which matches with demographic characteristics. Most of the studies don’t do this comparability cohort. This fact does not invalidate or diminish the importance of the data collected but is important at the moment the authors evaluate the scientific rigors of the articles.

Also, most of the papers did not mention if the diagnosis of skin cancer was confirmed by cutaneous biopsy and if these patients sought dermatological services outside their heart transplant centers. Both hypothetical situations can be a risk of bias in the outcome.

In the analysis of the quality of the studies using the Newcastle-Ottawa Quality Assessment Form for Cohort Studies modified, the poor and fair quality of 14 studies is considered also a limitation of this study.

### Additional comments

Fifty-five years have passed since the first human heart transplant was performed. During this period, the authors are able to draw a general overview of these patients in regard to skin neoplasm development.^52^ This systematic review shows that future research studies need to analyze exclusively heart transplant patients and specifically the outcome of skin cancer. It also reinforces the importance of taking care of this population in a multidisciplinary approach, where the dermatologist plays a key role.

Due to the complexity of the tailored immunosuppressive therapy and the environmental and individual risk factors, more studies are needed to understand this interaction.

The authors emphasize the importance of understanding ethnicity, accurately evaluating sunlight exposure, viral status, family history, comorbidities, and sunscreen use in the studies, and judiciously analyzing the different types of skin cancer, mainly SCC, BCC, MM. It is important to screen and identify which patients are at risk of developing skin cancer, and to act accordingly.

The authors reinforce the importance of focusing on each population of organ transplant, each immunosuppression therapy, and each malignancy, separately, providing the statistical analysis in a more precise and less generalized way. A more homogenous methodology will be helpful to understand the complexity of this cohort of patients.

## Conclusion

In conclusion, the risk of post-transplant NMSC and melanoma appears to be increasing universally, and heart transplant patients have a high frequency of skin cancer and other skin conditions. Such measures and well comprehension may prevent many skin cancers in this population.

Healthcare professionals should be highly familiarized with each regional organ transplant program and its demographic characteristics to improve the outcome of skin cancer among transplant patients.

This study reinforces that the dermatologist plays an important role in the short and long-term follow-up of heart transplant patients. This population requires a careful and continuous evaluation from the dermatologist. This approach can change morbidity, mortality, and the quality of life of many patients after the heart transplant. This study shows that improving the communication between transplant surgeons, transplant cardiologists, and dermatologists is a crucial and essential action in the care of heart transplant patients.

## Financial support

None declared.

## Authors’ contributions

Nathalia Hoffmann Guarda Aguzzoli: Conception and study; data collection, or analysis and interpretation of data; statistical analysis; drafting the article or critically reviewing it for important intellectual content; obtaining, analyzing, and interpreting data; effective participation in research guidance; critical review of the literature; final approval of the final version of the manuscript.

Ana Laura Bueno: Conception and study design; data collection, or analysis and interpretation of data; statistical analysis; drafting the article or critically reviewing it for important intellectual content; final approval of the final version of the manuscript.

Yağmur Halezeroğlu: Data collection, or analysis and interpretation of data; statistical analysis; obtaining, analyzing and interpreting data; effective participation in research guidance; final approval of the final version of the manuscript.

Renan Rangel Bonamigo: Conception and study design; data collection, or analysis and interpretation of data; statistical analysis; drafting the article or critically reviewing it for important intellectual content; obtaining, analyzing and interpreting data; effective participation in research guidance; critical review of the literature; final approval of the final version of the manuscript.

## Conflicts of interest

None declared.
